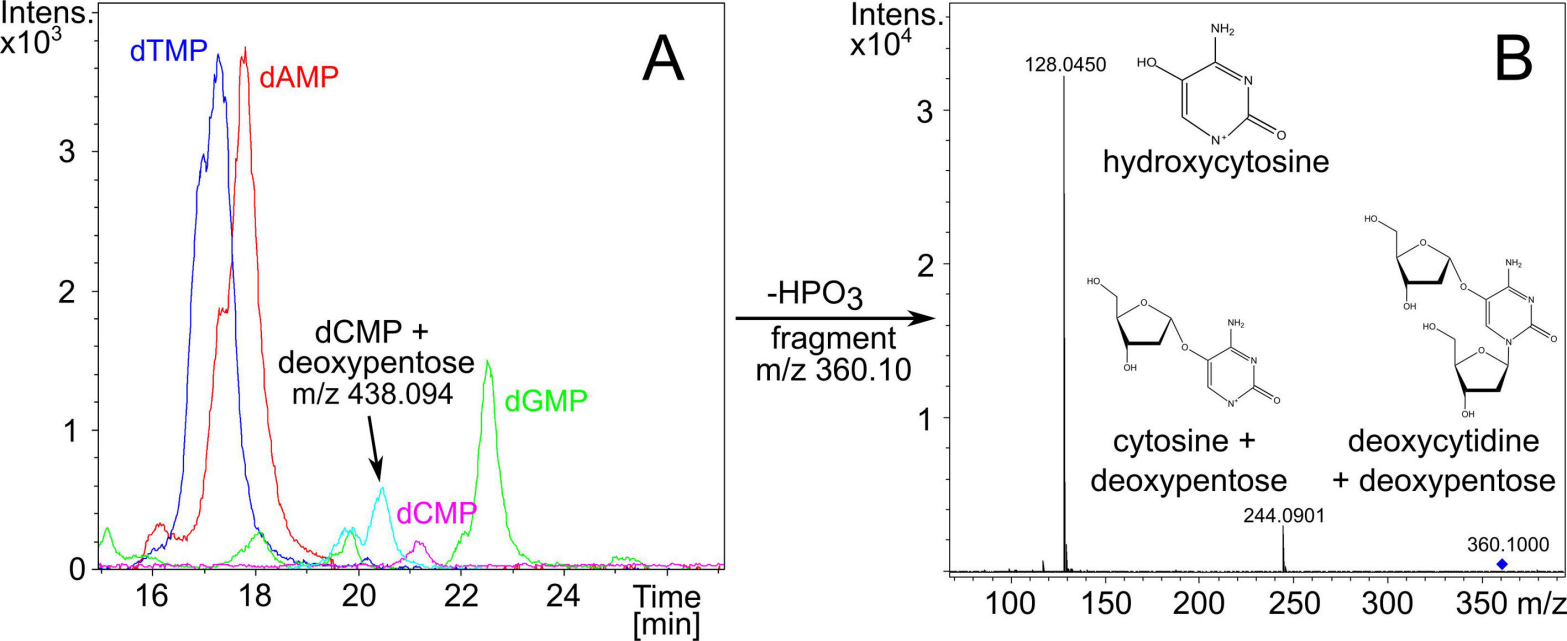# Articles of Significant Interest in This Issue

**DOI:** 10.1128/aem.00047-26

**Published:** 2026-01-27

## SCIENCE LOST IN TRANSLATION

This editorial by *Applied and Environmental Microbiology* (AEM) editor in chief Gemma Reguera (e02229-25) explores the innate and acquired nature of linguistic bias and its negative impacts on scientific progress, highlighting efforts at AEM to address this issue in academic publishing.


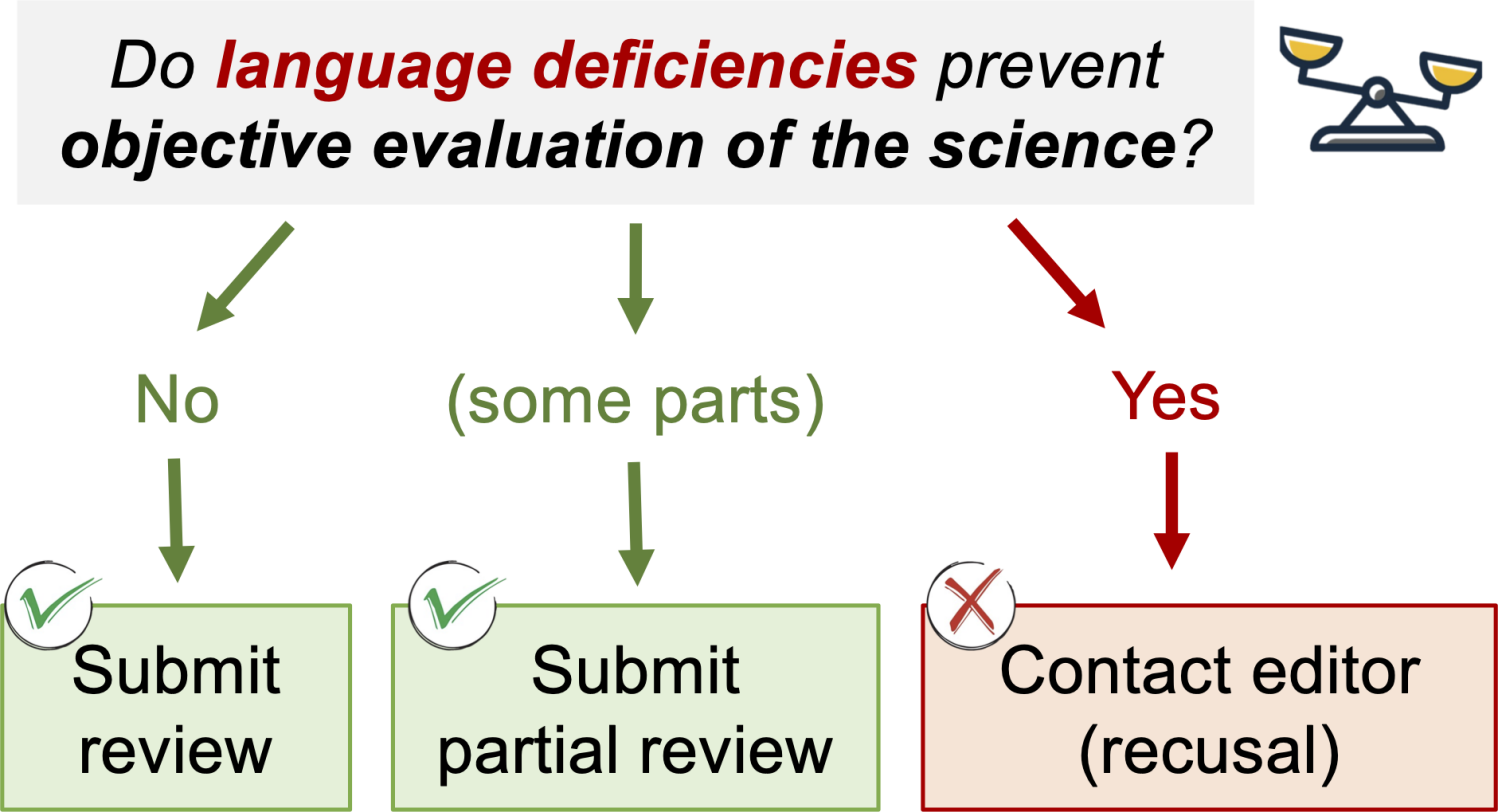


## A CRISPR’ED REVIEW OF BIFIDOBACTERIA

A timely minireview by Shin and Barrangou (e1703-25) about the occurrence and applications of CRISPR-Cas systems in bifidobacteria, key members of the human gut microbiota with probiotic potential.


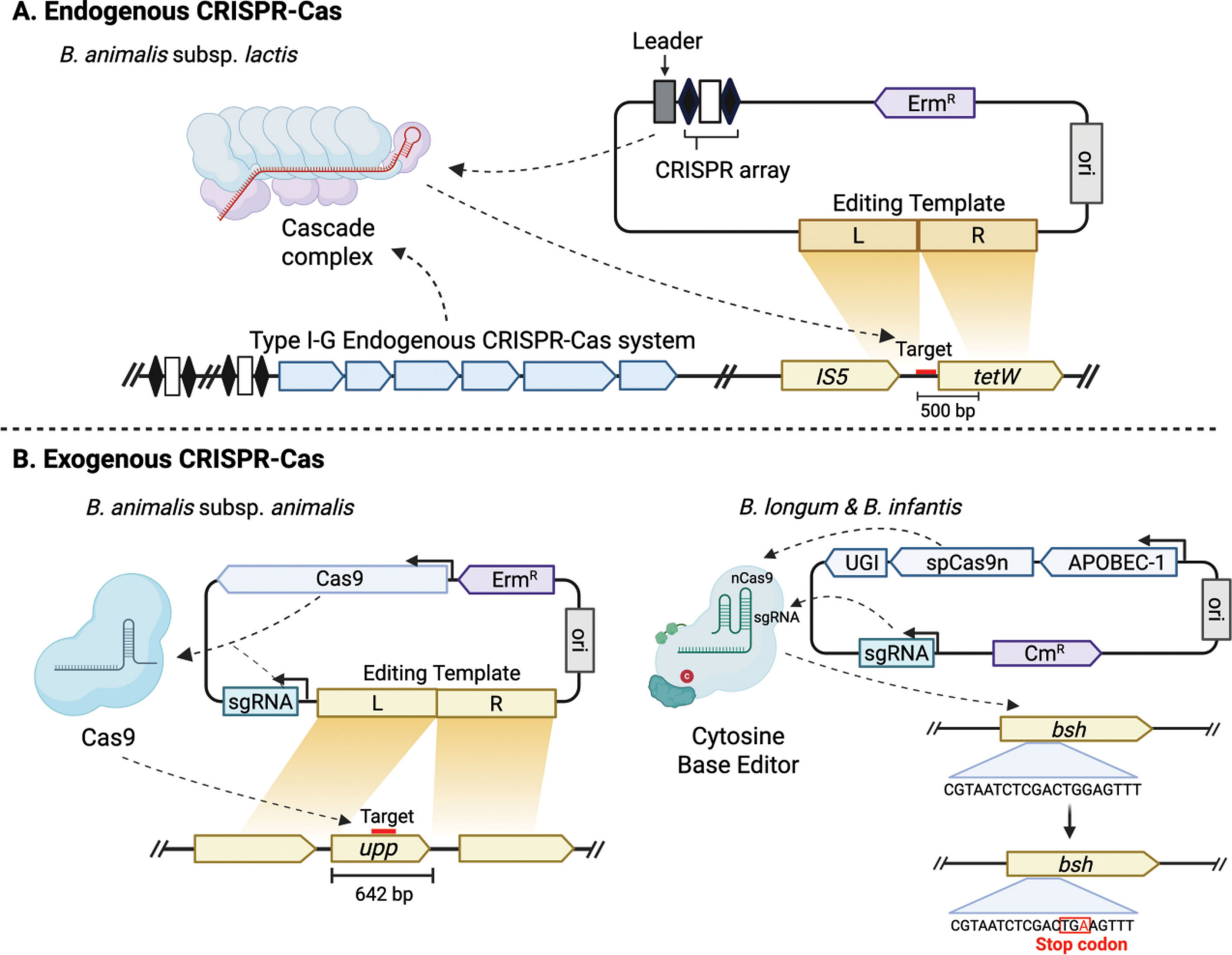


## THE “PLASMID PARADOX” UNDER REVIEW

Despite fitness costs, antimicrobial resistance plasmids are not eliminated by selection. This minireview by Hevar N. Abdulqadir (e01983-25) explores this paradox by examining the many factors that dynamically modulate the benefits and costs of plasmid carriage and, by extent, the threat of mobile resistance.


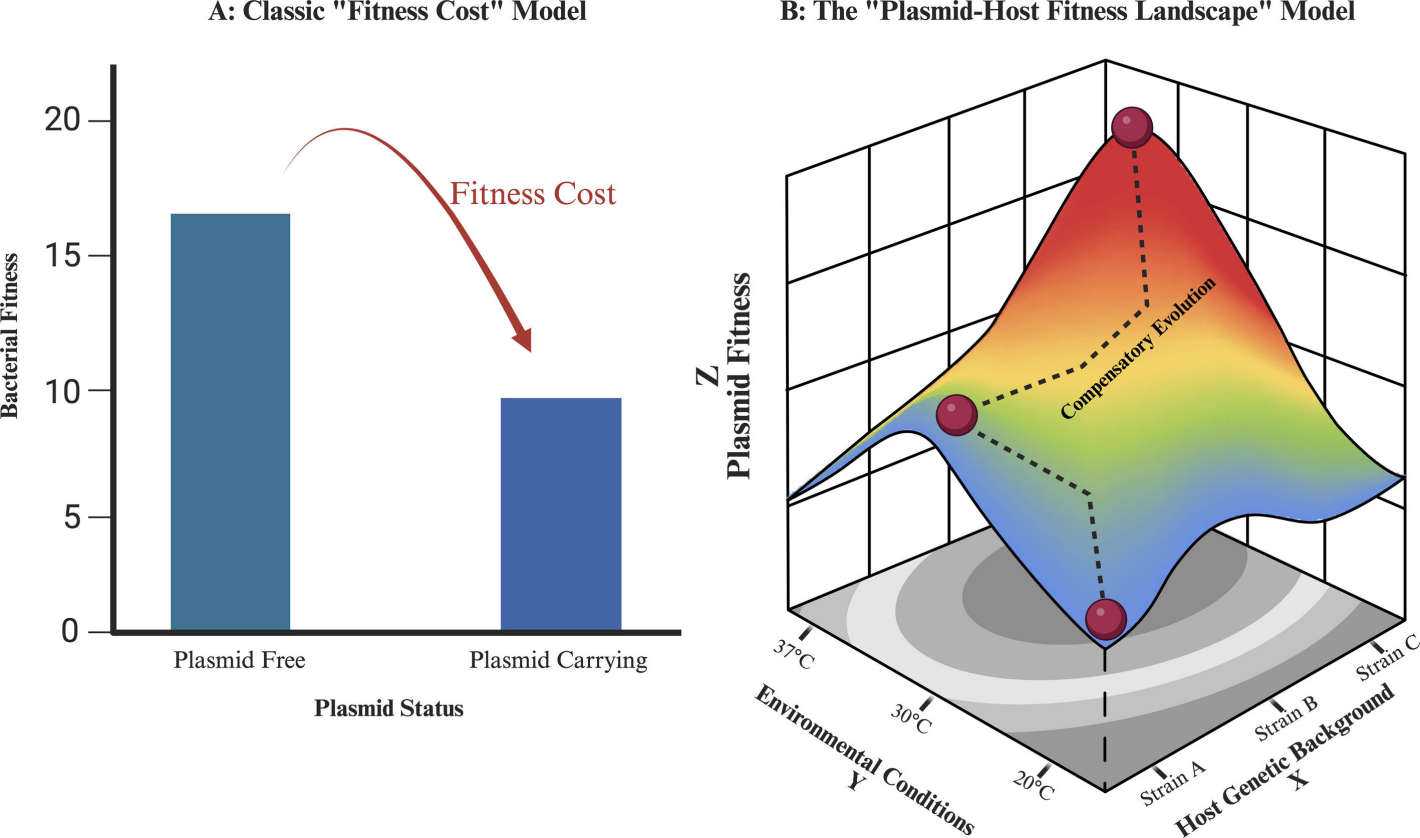


## ENZYME ENGINEERING HEATS UP

Yang et al. (e01860-25) combined automated computational design with structure-based rational design to engineer a subtilisin-like protease C2 with keratinolytic activity at temperatures near or above 100°C and resistance to polyextreme conditions relevant to feed, food, and leather industries.


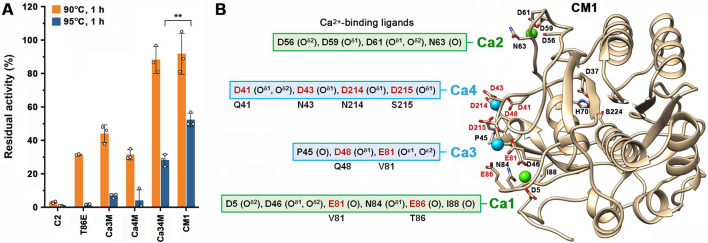


## A PINCH OF LIME TO RECOVER ACIDIFIED SOILS

Lime amendments are used to recover acidified soils and increase forest resilience to climate change. Hosmer et al. (e02171-24) describe changes in the bacterial/archaeal communities of the upper forest floor that may serve as an early indicator of soil recovery in response to liming.


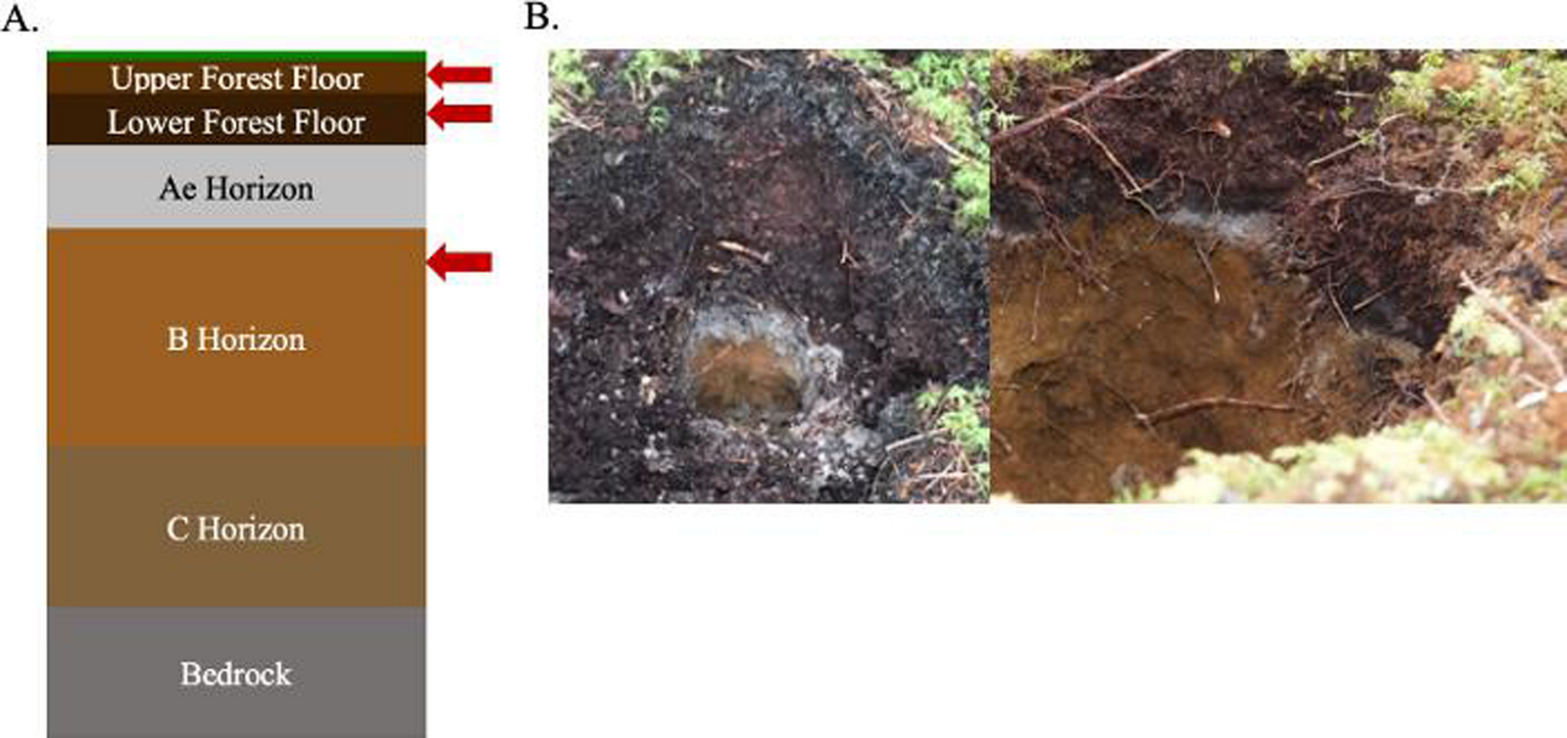


## A PENTOSE TRICK FOR PHAGE EVASION OF HOST RESTRICTION

Brandt et al. (e01333-25) describe a novel mechanism for phage DNA modification and evasion of host restriction defenses: attaching a deoxypentose to cytosine does the trick.